# Designing Expandable-Structure Robots for Human-Robot Interaction

**DOI:** 10.3389/frobt.2022.719639

**Published:** 2022-04-11

**Authors:** Hooman Hedayati, Ryo Suzuki, Wyatt Rees, Daniel Leithinger, Daniel Szafir

**Affiliations:** ^1^ Department of Computer Science, University of Colorado, Boulder, CO, United States; ^2^ Department of Computer Science, University of Calgary, Calgary, AB, Canada; ^3^ ATLAS Institute, University of Colorado, Boulder, CO, United States; ^4^ Department of Computer Science, University of North Carolina at Chapel Hill, Chapel Hill, NC, United States

**Keywords:** deployable robot, human-robot interaction, modular robot, origami robotics, deployable structures, shape-changing robots

## Abstract

In this paper, we survey the emerging design space of expandable structures in robotics, with a focus on how such structures may improve human-robot interactions. We detail various implementation considerations for researchers seeking to integrate such structures in their own work and describe how expandable structures may lead to novel forms of interaction for a variety of different robots and applications, including structures that enable robots to alter their form to augment or gain entirely new capabilities, such as enhancing manipulation or navigation, structures that improve robot safety, structures that enable new forms of communication, and structures for robot swarms that enable the swarm to change shape both individually and collectively. To illustrate how these considerations may be operationalized, we also present three case studies from our own research in expandable structure robots, sharing our design process and our findings regarding how such structures enable robots to produce novel behaviors that may capture human attention, convey information, mimic emotion, and provide new types of dynamic affordances.

## 1 Introduction

The ability to dynamically change shape and size is a key evolutionary advantage for many biological organisms. For example, pufferfish (Tetraodontidae) and the frilled lizard (*Chlamydosaurus kingii*) can change size as a self-defense mechanism, with the pufferfish able to expand up to three times their original size to warn predators and the frilled lizard able to expand a large frill around its neck, which is folded most of time, when threatened. Other organisms use size and/or shape changes for different purposes. For instance, male magnificent frigatebirds (*Fregata magnificens*) inflate their red throats to attract females, while octopuses change their structures to adapt to dynamic changes in their environment or interact with particular objects. Several fields have adapted this idea and developed shape-changing structures as solutions to various engineering challenges, leading to innovations in the automobile industry (e.g., roof structures in convertible cars), architecture [e.g., temporary exhibition rooms (Escrig and Valcarcel, 1993)], and design (e.g., self-inflating life vests). In addition, human-computer interaction (HCI) researchers have investigated shape-changing properties for developing new types of physical user interfaces (Rasmussen et al., 2012).

**FIGURE 1:**
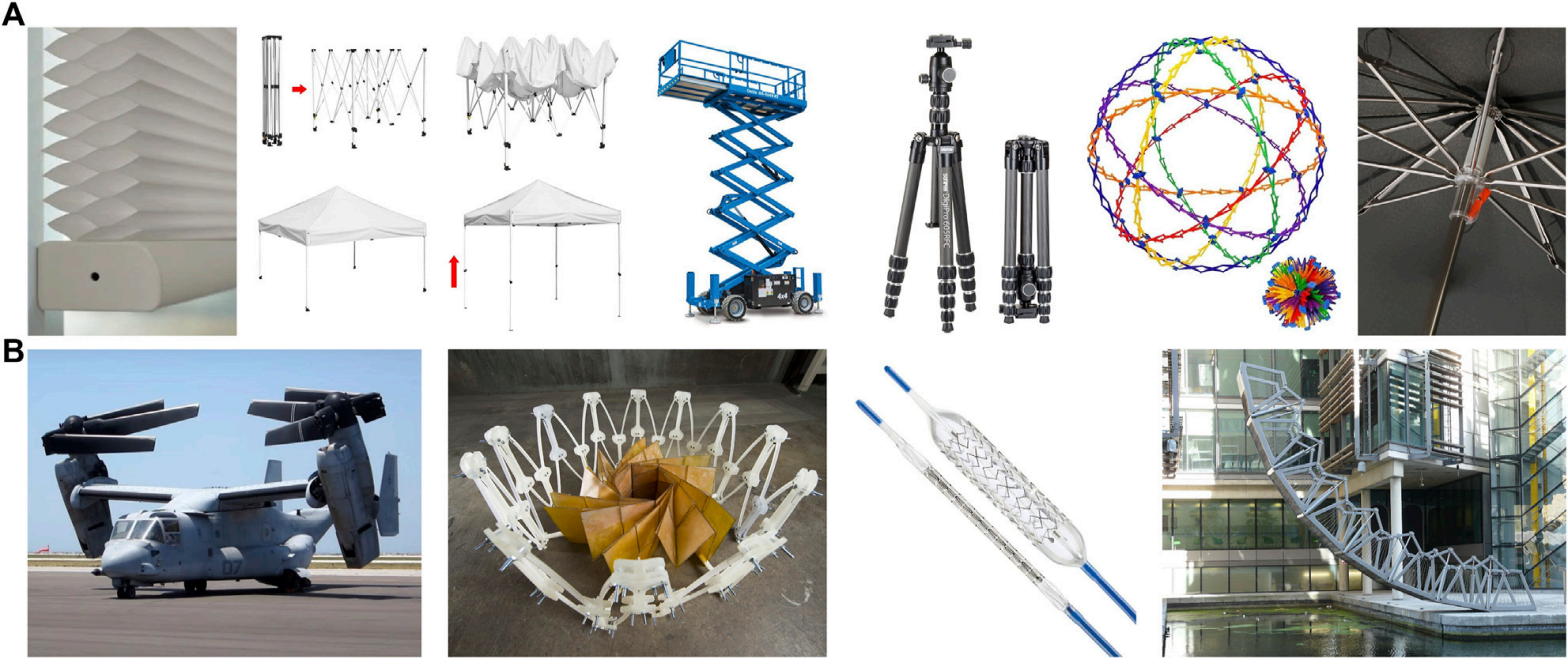
Expandable structures are found in a variety of everyday items, such as window shades, canopies, construction equipment, tripods and stands, toys, and umbrellas **(A)**. Expandable structures are also used in a variety of industrial and scientific purposes, including foldable aircraft, satellite design, medical devices, and architecture **(B)**.

Many of these systems can be described as *expandable structures*: ^1^ constructions that can change shape, and size using various linkages and joints (Pellegrino, 2002). Many expandable structures can be found in nature, such as in the leaves of hornbeams, flower petals, and the hind wings of beetles (Vincent, 2000; Wang et al., 2017). Engineered expandable structures, a subclass of more general shape-changing technologies, are present in a variety of common consumer products, such as umbrellas, Hoberman spheres, and Origami. Such structures are also used in a diverse set of industrial and scientific equipment, including various forms of construction cranes, stents and other medical devices, foldable satellites, and certain architectural designs, as in adaptive and morphing building structures (Del Grosso and Basso, 2010). Figure 1 illustrates the diversity of expandable structure, showcasing their applications across everyday and specialized items. There are several methods for changing the size and shape of expandable structures, including mechanical mechanisms (e.g., scissor assemblies, bistable structures, isokinetic/Hoberman mechanisms, etc.), pneumatic or hydraulic mechanisms (e.g., inflatable structures), or through thermal or electrical stimulation of certain materials [e.g., shape memory polymers (Liu et al., 2014)].

**FIGURE 2:**
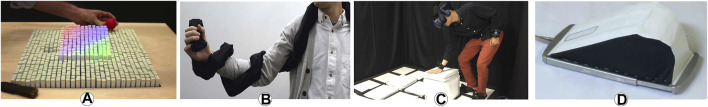
Examples of various ways shape-changing interfaces researchers and designers have proposed to augment human-computer interaction: **(A)** dynamically actuated shape displays such as Materiable (Nakagaki et al., 2016b), **(B)** deformable, actuated linkages such as LineFORM (Nakagaki et al., 2015), **(C)** inflatable structures such as TilePoP (Teng et al., 2019), and **(D)** Inflatable Mouse: a volume-adjustable mouse with air-pressure-sensitive input (Kim et al., 2008).

In this work, we are primarily interested in expandable structures and related shape-changing technologies in the context of human-robot interaction (HRI) research and applications, including interface technologies, haptics, visualization, and robotics. For instance, one of the primary uses of expandable and shape-changing structures from user interface research has been the development of novel technologies that provide users with physically dynamic interfaces [Figure 2, see (Alexander et al., 2018) and (Rasmussen et al., 2012) for full survey of this space]. The goals of such research have strong alignment with many traditional goals of HRI, where shape-changing technologies have been applied to develop devices that can adapt to users and the environment in new ways, communicate information to users, and/or provide novel, adaptive affordances. As an example, researchers have designed multi-touch display surfaces, where each touch point can be deformed to be convex, flat, or concave (Stevenson et al., 2010). This expandable surface matches the physical shape of the display to its visual counterpart, enabling more intuitive interactions, and we can envision HRI researchers applying similar methods to developing novel robot interfaces for teleoperation or supervision. Beyond such physical interfaces, expandable structures have also been used to create brain-computer interfaces (BCIs), which are also being explored for robotics. In (Jiang et al., 2020), expandable fiber probes adapt to contact various parts of nearby brain tissue, enabling scanning of a greater area of brain tissue with fewer surgical insertions and reducing patient risks in such procedures.

Another major focus of shape-changing structure research relevant to human interaction has been developing new forms of haptic feedback interfaces, particularly for use with Virtual Reality (VR). This research generally leverages expandable structures to provide *encountered-type haptics*, in which certain aspects of the surrounding real-world environment shift dynamically to provide physically resistive forces when users make contact with virtual objects. For example, FEELEX (Iwata et al., 2001) and shapeShift (Abtahi and Follmer, 2018; Siu et al., 2018) implemented dynamically actuated shape displays using an array of actuators in combination with a flexible screen and electro-active polymers, respectively. These devices provide the capability to simulate varying surfaces and shapes in VR. At a larger scale, TilePoP (Teng et al., 2019) and LiftTiles (Suzuki et al., 2020b) investigated the use of inflatable actuators to dynamically change the user’s surrounding physical environment to provide haptic proxies. Each of these haptic displays utilize expandable structures to create a VR experience that users perceive as more realistic, as the visual cues associated with virtual object interaction can be accompanied by real forces.

Recent work has also shown the potential of expandable structures for several other interaction-focused applications, such as visualization, design, and education. For example, the HATs (Mi and Sugimoto, 2011) and G-Raff (Kim and Nam, 2015) systems used height-changing structures to synchronize the height of objects with digital content on a tabletop surface, enabling intuitive interaction with 3D spacial data. LineFORM (Nakagaki et al., 2015) demonstrated how a physical line made from actuated linkages can transform into a wristwatch, a phone, and several other objects. This type of dynamic physical display allows for richer interactions with a wide array of objects and data. Additionally, it can provide new constraints on user interactions in order to provide guidance, presenting opportunities for the device to scaffold learning. Moreover, highly extendable linear actuators can achieve both shape- and size-changing transformations (Takei et al., 2012; Hammond et al., 2017; Hawkes et al., 2017). These devices present opportunities in several domains, ranging from enabling dynamic and self-erecting architecture to providing increased mobility in search and rescue operations by supporting adaptation to irregular terrain. The Topobo (Raffle et al., 2004) and ShapeClip (Hardy et al., 2015) structures allow a designer to construct different geometries of shape-changing interfaces and have shown potential for enhancing early education by helping children learn about relationships between physical formations and physical properties, such as balance and leverage.

In this paper, we focus on the integration of expandable structures and robotics as a promising avenue for improving human-robot interaction (HRI). In recent years, researchers and engineers have leveraged expandable structures for several robotic applications (Felton et al., 2014; Kornatowski et al., 2017; Perez-Guagnelli et al., 2018). For example, expandable structures have helped aerial robots navigate through narrow spaces (Falanga et al., 2018) and robot arms reach confined areas (Shikari and Asada, 2018). However, such prior deployments of expandable structures for robotics have primarily focused on specific aspects of robot task and/or control (e.g., manipulation, locomotion, etc.). In this paper, we instead categorize a broad range of HRI-relevant factors and implementation considerations while synthesizing several interaction-based use-cases for expandable structures and shape-changing robots. We use these categorizations as part of detailing an incipient design space in how such technologies may improve robot interactions with collocated humans.

As examples of this design space, we also highlight three specific implementations of expandable structures for HRI from our own research, including *RoomShift*, a ground robot that uses expandable structures to move furniture in a room in order to provide haptics for a human working in virtual reality (Suzuki et al., 2020a), *PufferBot*, an expandable structure for aerial robots that can improve safety while also introducing a new signaling mechanism to communicate with nearby humans (Hedayati et al., 2020), and *ShapeBot*, a miniature tabletop robot that can change shape individually and also as part of a larger ShapeBot swarm to convey various information to users (Suzuki et al., 2019b).

## 2 Expandable Structures for Robotics

To date, there has been very little work exploring robots with expandable structures from a human-robot interaction perspective. Instead, most prior work has focused on the mechanical aspects of building expandable structure robots, which fall within a broader category of shape-changing robots. While precisely classifying the full space of shape-changing robots is challenging, as some robots might cross categorical boundaries, systems developed in prior research generally fall into one of the following major groups: **modular** self-reconfigurable robots, **origami**-like robots, **tensegrity** robots, **soft** robots, or deployable/**expandable** robots.

Modular robots are robots made of identical or similar elements that can be attached in different ways to form different group structures (Støy, 2015; Shang et al., 2018). The Reconfigurable Modular Manipulator System (RMMS) (Kelmar and Khosla, 1990) was one of the earliest modular robots and consisted of a set of modular links and joints of various sizes that could be reconfigured according to specific tasks. This system introduced dynamism to industrial robotics, enabling faster productivity and reduced costs. In a similar vein, a modular isomorphic master-slave robotic system was developed (Zheng et al., 2013) to enable master robots to be highly adaptable to varying structures and degrees of freedom in slave robots, further increasing productivity and reducing costs in a wider range of domains. Chain-type robots, such as PolyPod (Yim, 1994), CONRO (Castano et al., 2000), and PolyBot (Duff et al., 2001), are robots constructed as a connected series of modular parts, simplifying and expanding the level of dynamic abilities that robots can achieve. Lattice-type robots, such as Molecule (Kotay et al., 1998) and 3-D-Unit (Murata et al., 1998), are constructed as a grid-like network structure of modular pieces. These robots provide similar benefits as chain-type robots, while also expanding their reconfigurability into another dimension in space. Hybrid modular robots, such as M-TRAN III (Kurokawa et al., 2008) and SuperBot (Salemi et al., 2006), use a combination of chain- and lattice-type structures. Changibles (Roudaut et al., 2014) and Cubimorph (Roudaut et al., 2016) are shape-changing robots that leverage a modular and reconfigurable design to achieve different geometries, allowing for richer and more intuitive interactions with dynamic shape displays. ChainFORM (Nakagaki et al., 2016a) integrates modular sensing, display, and actuation to further enhance interactions.

Another category of robots that exhibit shape- and/or size-changing properties are Origami-like robots. Origami has been used in many engineering areas (Okuzaki et al., 2008; Ma and You, 2013) and is increasingly feasible for robotics due to improvements in fabrication and actuator technologies. Examples of origami-like robots include robotic sheets that can be folded into different morphologies (Hawkes et al., 2010) and a set of programmable triangles which can create different patterns (Belke and Paik, 2017). Origami robots offer several advantages, including the elimination of redundant materials used in separate tasks, reducing the amount of materials needed overall, and their foldable designs may often serve dual purposes, such as providing a robot chassis with built-in protection [e.g., as in origami-inspired mechanisms for aerial robots (Kornatowski et al., 2017; Sareh et al., 2018; Shu and Chirarattananon, 2019)]. To date, most research on Origami robots has focused on physical design and actuation (Lee et al., 2013; Onal et al., 2014; Vander Hoff et al., 2014; Miyashita et al., 2015) or on using smart materials to create self-folding robots (Paik et al., 2010; Paik and Wood, 2012; Tolley et al., 2014; Firouzeh and Paik, 2015). Recently, researchers have also explored Kirigami structures, an extension of Orgami that supports cutting in addition to folding, for deployable robot design (Sedal et al., 2020).

Tensegrity robots and soft robotics take a different approach towards developing actuated systems. Tensegrity robots focus on designing systems made of tensegrity structures (Snelson, 1965), which is an abbreviation of *tensile integrity*. Tensegrity robots are typically formed from constructions of ropes, tube, springs, and joints that provide strength and compliance while being lightweight. As a result, tensegrity robots have particular relevance to space robotics (SunSpiral et al., 2013; Sabelhaus et al., 2015). Currently, most research in tensegrity robotics is focused on design, locomotion, and control (Caluwaerts et al., 2014; Sabelhaus et al., 2015; Zhang et al., 2017; Vespignani et al., 2018; Wang et al., 2019). To the best of our knowledge, such structures have yet to be explored from a human-robot interaction perspective.

In contrast to rigid systems, soft robots actuate elastic and compliant materials, such as rubbers, hydrogels, and silicone elastomers (Coyle et al., 2018). There are a variety of actuation methods for soft robots, including pneumatic, electroactive polymer, tendon driven, shape memory alloy, and electro- and magneto-rheological materials (Das and Nabi, 2019). From a HRI perspective, soft robots may improve safety in collocated use cases due to their complaint nature and have been explored for several applications, including wearable robots that provide human movement assistance (Maeder-York et al., 2014; Park et al., 2014) or convey emotions (Hu and Hoffman, 2019).

In this paper, we are particularly focused on a subclass of shape-changing robots: expandable (i.e., deployable) structure robots that use rigid mechanisms to change their size and shape to improve mobility or gain new interactive capabilities. In terms of mobility, various “reconfigurable” or “hybrid” ground-mobile robots have been developed that may change form to use either wheel or leg locomotion to adjust to changes in terrain [e.g., (Ding and Xu, 2009; Chen et al., 2013; Reid et al., 2020); for a survey, see (Russo and Ceccarelli, 2020)]. Alternatively, the AmphiHex-I presents a design for an amphibious robot with leg-flipper composite propulsion, enabling the robot to walk and move under water (Liang et al., 2012). Such concepts have also been explored in aerial systems, where researchers have created foldable drone frames to enable navigation through confined spaces (Falanga et al., 2018) and hybrid systems, such as HeritageBot, capable of walking and flying (Ceccarelli et al., 2018). Beyond mobility, researchers have used expandable structures for robots in various ways to enable dynamic robot re-sizing. For example, expandable structures have led to deformable wheels for robots (Lee et al., 2013), robots capable of self-folding from a sheet to a 3D structure (Miyashita et al., 2015), and robots arms able to extend to gain additional manipulation reach (Shikari and Asada, 2018). While promising, such research typically details the design of one particular expandable structure robot or application. To help researchers seeking to explore expandable structures for HRI, below we synthesize several implementation considerations for developing expandable structures specifically within the context of robotics and describe a broader design space regarding how expandable structures may afford new methods of interaction between humans and robots.

## 3 Implementation Considerations

In this section, we present an overview of various implementation details necessary for developing expandable structures for HRI research. To help future researchers and designers better reason through the various alternatives and opportunities available for developing expandable structure robotics, we describe considerations involving expandable structure type, actuation method, and integration with robot platforms. Figure 16 provides a visual reference for these implementation considerations and potential interaction goals, which are detailed in Section 4, and shows the decisions we made in each of our case studies described in Section 5.

### 3.1 Expandable Structure Types

Depending on the purpose of the device, expandable structures may use various methods to change their shape and size. While several methods for classifying expandable structures have been proposed [see (Fenci and Currie, 2017) for a survey], we are primarily interested in two main categories: those that utilize mechanical joints and those that utilize the physical properties of continuous materials. One of the most widely-used mechanical methods of expansion is a scissor-like structure [Figure 4A, see (Zhao et al., 2009) for a review of the mechanics underlying scissor structures]. Most commonly, these structures allow for linear expansion and retraction, an example of which is the electric scissor lift. However, scissor-like structures may also be used to expand in a radial fashion. Another common structure used for expansion is the Hoberman linkage (Figure 4B). This structure is comprised of a similar series of parts as the scissor-like structure, but instead allows for radial expansion. Six Hoberman linkages may be aligned according to the edges of an icosidodecahedron and actuated simultaneously in order to create a Hoberman sphere. Another type of expandable structure that is common among consumer products are those that use retractable plates, such as a camera shutter or movable form of wheelchair ramp used on buses to provide for wheelchair access. A similar concept is found in telescopic structures, which use concentric tubular sections that slide into one another (See Figure 4D). Another class of mechanical expandable structures are reel-based structures (Hammond et al., 2017; Suzuki et al., 2019a; Suzuki et al., 2021) or those that use revolute joints to unravel chain- or lattice-type structures (Figure 4C). These structures are unique in that they can allow for expansion in all three dimensions of space.

**FIGURE 3:**
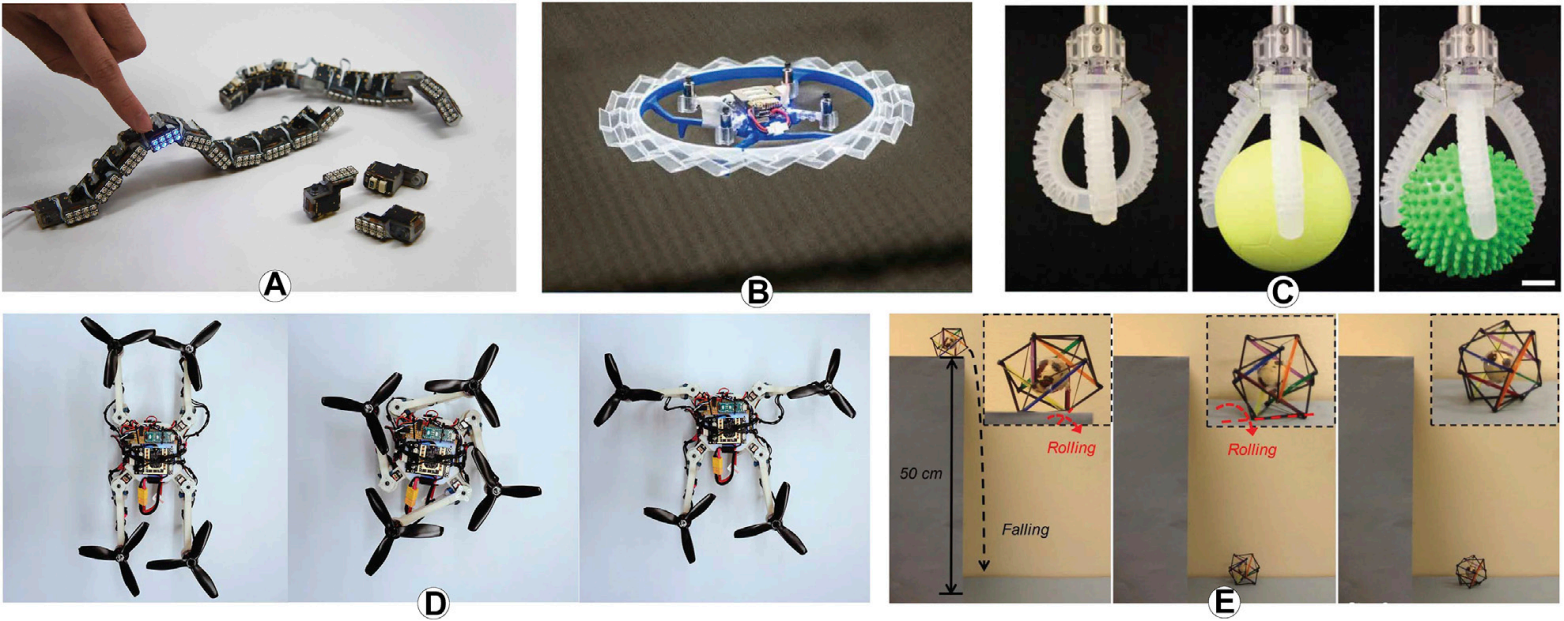
Examples of robots with shape-changing technologies, including **(A)** modular robots in ChainFORM (Nakagaki et al., 2016a), **(B)** origami structures in Rotorigami (Sareh et al., 2018), **(C)** soft materials (Truby et al., 2018), **(D)** deployable structures that enable folding, expansion, and contraction (Falanga et al., 2018), and **(E)** tensegrity systems (Wang et al., 2019).

**FIGURE 4:**
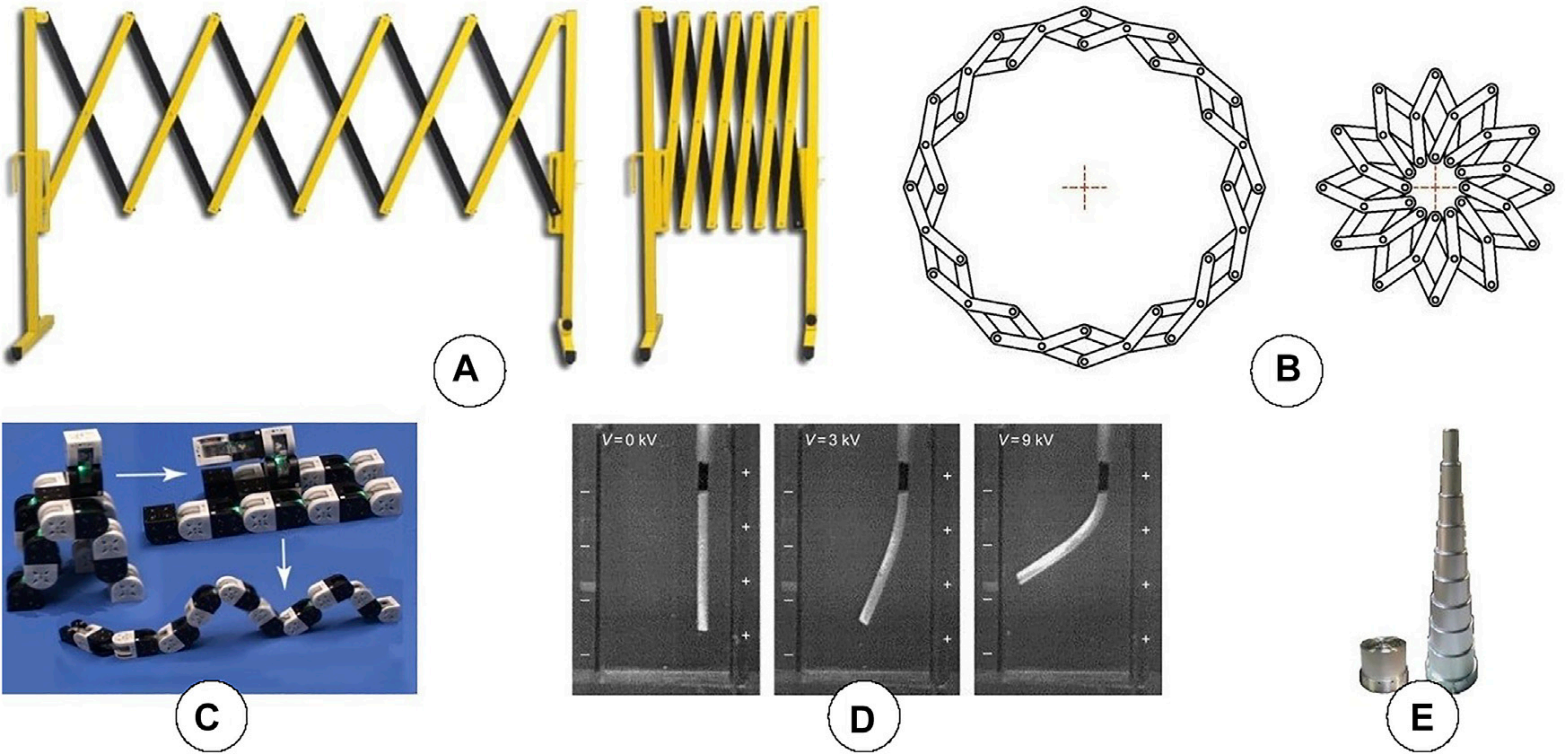
**(A)** A scissor-like expandable structure, capable of expanding linearly. **(B)** A Hoberman linkage, capable of expanding radially. **(C)** A chain-type expandable structure, capable of expanding various components in all directions in space (Kurokawa et al., 2008). **(D)** An electro-active polymer, which may be capable of forming different shapes. **(E)** A telescopic structure.

Expandable structures that utilize the physical properties of continuous materials do so with soft, flexible materials, such as silicone (Figure 4D). The benefit of these structures typically resides in their ability to take on many different types of shapes and curvatures. Typically, these structures form a 3D surface, which may be expanded and/or morphed into different shapes. For example, one study used a self-expanding silicone stent to help patients with esophageal cancer swallow food (Siddiqui et al., 2007). An example of shape-changing soft expandable structures from user interaction research is PneUI (Yao et al., 2013), which uses soft composite materials to create a shape-changing interface.

When designing an expandable structure, one must carefully analyze the physical domain in which the structure serves a purpose: How many dimensions does the structure need to expand into? How large must the structure be? How strong or rigid does the structure need to be? The answer to these questions will be a primary determining factor in deciding the type of structure that is best suited for the problem. For example, if a structure only needs to expand in one direction and must interact with heavy objects, a rigid, scissor-like structure is a natural choice. On the other hand, if the structure is intended to represent data in various forms or is intended to be touched and deformed by a human, a soft, shape-changing structure may be better suited.

### 3.2 Actuation Methods

There are several methods researchers may choose to actuate expandable structures, including hydraulic, pneumatic, electric, and mechanical. Hydraulic actuators consist of a hollow cylindrical tube along which a piston can slide. A hydraulic pump delivers a regulated flow of compressed liquid to move the piston. These actuators are capable of exerting forces of relatively high magnitude, but cannot achieve high acceleration compared to other actuators. Pneumatic actuators work in a similar fashion as hydraulic actuators, but instead use air pressure to move the piston. They can also provide forces of high magnitude with relatively small volumes of air, but require complex systems of components (compressors, reservoirs, filters, etc.) that may result in inefficient energy loss. Electric actuators typically convert the rotational force of an electric rotary motor into a linear movement, which can be done with hydraulic or mechanical mechanisms. Another form of electric actuators use a series of oppositely aligned magnets and electric coils driven in opposing phases to generate a linear force without extraneous mechanical or hydraulic components. Another type of electric actuation uses electro-active polymers, which act like artificial muscles. When an electric current is supplied through the polymer, it contracts (Novack et al., 2021). Releasing the current allows for the polymer to expand again. In this case, the actuation method may act as the expandable structure itself. Mechanical actuators convert rotational force into linear force through the use of components such as belts, screws, or gears. What makes the mechanical actuators different from electrical actuators is that, in mechanical actuators, the energy needed for actuation is stored in a non-electric way, such as in springs.

Different actuation methods provide trade-offs for researchers and designers seeking to create expandable structures for human interaction. For example, if the intended interaction may involve direct physical contact with humans, an actuation method that exerts relatively lower magnitudes of force may enhance user safety—in the case of a system malfunction, there is less potential for harm to the user. Conversely, if an expandable structure is used to alter or manipulate objects and/or the environment, as is the case with structures that enable encountered-type haptics, an actuation method capable of exerting higher magnitudes of force may be necessary. If the expandable structure is intended for visualization of various data, actuation precision or speed may be primary considerations.

### 3.3 Robot Integration

Integrating expandable structures with robots may require specific considerations of robot type, size, and capabilities. There are several different ways of classifying robots, such as considering morphology (e.g., anthropomorphic/human-like, biological/zoomorphic, or functional) (Fong et al., 2003; Li et al., 2010), capability (e.g., fixed-based manipulation, ground-mobile, ground-mobile manipulators, aerial), or degree of autonomy (Szafir et al., 2017). For traditional and pre-existing robot platforms, expandable structures may be added as sub-components [e.g., an expandable structure for compliant robot grippers as in (Kaur and Kim, 2019)] or as entire frames [e.g., a protective frame around a drone (Hedayati et al., 2020)]. Alternatively, new robots may be designed to leverage expandable structures as central components of the robot itself, as in the Triple Scissor Extender Robot Arm (Shikari and Asada, 2018), a new design for an expandable structure robot arm that supports manipulation in cluttered and confined areas. In both contexts, relevant considerations for roboticisits include power, weight, and structure materials. Power for expandable structures may be self-contained or draw on a central robot power supply, while weight and materials may be selected based on platform needs and application goals. For example, a structure made for manipulation or lifting of heavy objects would require a strong, rigid structure, while a structure made to reduce the impact of collisions would require a more compliant material to reduce the impact force. Prior work has explored expandable structures constructed with various materials, including metal (Shikari and Asada, 2018), ionic polymer-metal composite (Niu et al., 2015), soft silicon (Takei et al., 2011a), latex (Stevenson et al., 2010), plastics (Sedal et al., 2020), and other soft materials. Other promising materials that have yet to be extensively explored for expandable structures integrated with robots include wire structures, wood, and linen. Roboticists seeking to integrate expandable structures with existing platforms must additionally consider how to mount expandable structures in a manner that does not impede robot mobility or existing capabilities while ensuring visibility, potentially by leveraging universal mounting systems or developing custom mounting plates, as in (Suzuki et al., 2020a; Hedayati et al., 2020).

Across all types of robots with expandable structures, researchers must also consider size and expandable structure capabilities. The size of the expandable structure will likely increase with the size of the robot. As the size of the structure increases, so will its weight. With these correlations, structure material may be the primary consideration (i.e., robots with limited payload capacity must make use of lightweight materials for their expandable structures). A similar comparison can be made with smaller robots, which may only be able to support smaller payloads due to mounting challenges. Overall, researchers and developers will need to consider the trade-off space between weight and strength (resulting from material choice and structure design) and payload capacity. Additionally, larger robots that utilize expandable structures as a frame may need additional support mechanisms for the structure to prevent it from collapsing.

In addition to material choice, the expandable structure’s intended capabilities will have a large influence on the proper actuation choice for the structure. For example, if the purpose of the expandable structure is to enable better manipulation of potentially heavy objects, the actuation will need to be able to output a large force. In this case, a hydraulic or pneumatic actuator will likely be a good choice. In some special cases, an expandable structure that is expected to endure high forces may be able to use less powerful actuators. For example, if the structure is intended to protect the robot from collisions, one could rely on other mechanisms besides the actuators to prevent the structure from collapsing upon collision. One such mechanism could be pieces of the structure that lock in to place upon expansion of the structure, much like locking one’s knees when fully straightening one’s legs. On the other hand, if the purpose of the structure is to enhance fine manipulation or to visualize data, a more precise actuation method (e.g. electromechanical) may be required.

## 4 Interaction Design Space

**FIGURE 5:**
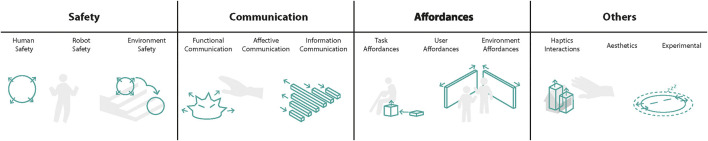
Expandable structures open up new design spaces for human-robot interactions by promoting physical safety, providing novel communicative channels, supporting adaptive affordances, and altering experiential aspects such as aesthetics or enjoyment.

In this section, we describe the design space regarding how expandable structures may be integrated with robotic systems to improve human-robot interaction (Figure 5). We highlight how expandable structures may provide robots with new ways to interact with both their surrounding environment and collocated humans, expand robots abilities to signal and convey information to humans, improve human-robot safety, and affect experiential aspects of interactions, such as altering aesthetics or enhancing enjoyment, curiosity, or playfulness.

**FIGURE 6:**
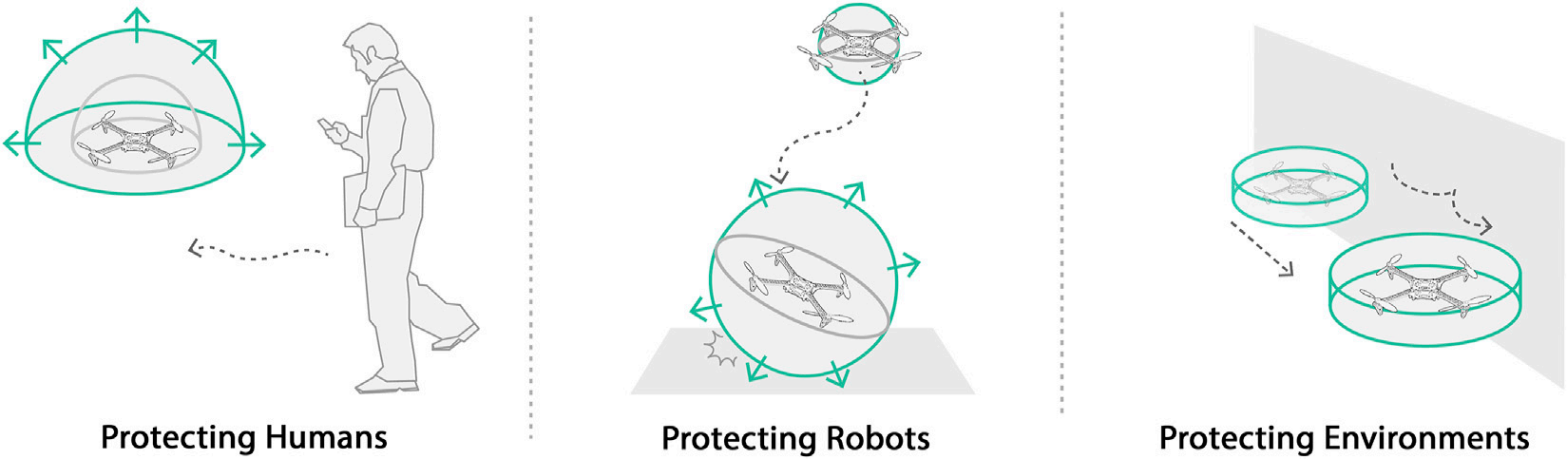
Robots with expandable structures can improve the safety of collocated humans, the robot itself, and the environment.

**FIGURE 7:**
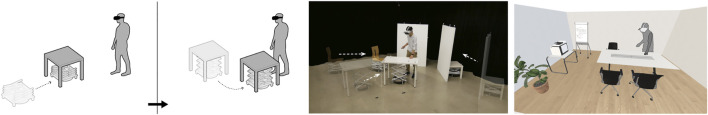
RoomShift is comprised of a swarm of shape-changing robots that provide haptic feedback in VR by manipulating physical furniture and walls. Multiple robots move environmental objects to collectively construct and adapt a physical haptic environment that matches virtual scenes. Above, we show the physical environment with a corresponding virtual scene, with a human silhouette added for a reference.

### 4.1 Adaptive Affordances

Providing robots with the ability to adapt their shape and size based on interaction context opens up many new possibilities in how robots may interact with humans, objects, and their environment. Such adaptation may be related to a specific HRI *task*, where, for example, expandable structures may afford a collaborative or teleoperated robot with new capabilities for manipulating or assembling objects (e.g., altering leverage to adjust objects that would otherwise be too unwieldy or expanding to grasp otherwise out-of-reach objects), new ways to navigate through confined environments that would otherwise be infeasible to operate within, or new ways for multiple robots to work together by combining expandable structures in a team fashion, making use of the fact that many types of expandable structures are modular in design. Alternatively, adaptation may be related to the *user*, where, from a human’s point of view, expandable structures might change shape and/or size to indicate different possibilities for user interaction (e.g., a robot that detects an internal fault might change shape to enable a technician easier access to internal components that an expandable structure would guard in normal circumstances). Certain applications may involve adaptation to both task and user, as in the design of expandable structure robotic exoskeletons [e.g., (Li et al., 2019)], where expandable structures may provide singularity-free joints for wearable robots that do not compromise human limb function (Castro et al., 2019). Expandable structures also offer new capabilities for user control of robots, particularly for novices, who may lack the situational awareness or experience necessary to accurately control complex systems such as redundant manipulators or aerial robots leading to self-collisions, crashes, and/or damage to surrounding objects or the environment. Expandable structures may offer a new way in which robot operators may physically “probe” the surrounding robot environment in a safe manner by bumping into other objects, walls, ceilings, etc. without damage. Such an interaction may be used in educational or training scenarios, where users gain confidence and abilities controlling new robotic systems or in real systems, potentially combined with haptic feedback controls, to enhance user awareness of the robot environment. In a similar fashion, expandable structures can enable robots to work with users in new environments that were previously too cluttered or confined (Shikari and Asada, 2018; Hedayati et al., 2020).

One particularly promising application of how expandable structures may provide robots with adaptive, task-based capabilities to support human interaction is through using robots as novel haptic interfaces, especially in conjunction with virtual reality. As described in Section 2, encountered-type haptics focuses on providing users with physically resistive forces that simulate virtual objects to enhance user presence in VR. With the ability to change size and shape, a single robot might be able to represent several different sizes or types of objects in a virtual environment. For example, a VR user could interact with several virtual balls of different sizes that are all physically represented by a single robot with a Hoberman sphere structure that expands or contracts to match the size of the ball used at any given time. We detail our own work at the intersection of expandable structure robots and encountered-type haptics for VR in Section 5.1.

### 4.2 Non-Verbal Communication

One of the ways that we can improve human-robot interaction is by expanding the communication mechanisms available for exchanging information between humans and robots. Prior research has explored a variety of communicative channels, including gaze (Mulu, 2006; Andrist et al., 2012; Andrist et al., 2014; Admoni, 2016; Oliveira et al., 2018), implicit motion (Dragan et al., 2013; Szafir et al., 2014; Sadigh et al., 2016; Zhou et al., 2017; Kwon et al., 2018), gesture (Waldherr et al., 2000), sound (Cha and Matarić, 2016; Cha et al., 2018a), visual displays, lights (Szafir et al., 2015; Baraka et al., 2016; Song and Yamada, 2018), haptics (Guerreiro et al., 2019; Guinness et al., 2019), projection (Pierce et al., 2012; Cauchard et al., 2019), and augmented reality (Hedayati et al., 2018; Walker et al., 2018; Cao et al., 2019; Szafir, 2019; Walker et al., 2019). Expandable structures represent a promising new signalling medium to add to this collection of methods for supporting human-robot information exchange, which may take the form of functional cues regarding the robot and task or affective signals that communicate emotional information.

#### 4.2.1 Functional Communication

We envision that expandable structures may be used to convey a variety of common functional signals to collocated users, including information about internal states (e.g., expansion of the structure might correlate with battery level), higher-level information about processes or tasks (e.g., the percentage of completion of a task), or goals and intent (e.g., expanding a structure the direction the robot intends to move and contracting a structure on the opposite side). Expandable structures might also enhance other methods of signaling in HRI by increasing or decreasing the size of other signaling devices. For example, LED arrays or strips can be flashed in different patterns to signal information to humans. However, the discrenability of such visual signals depends on the distance between the robot and the user and the distance between individual lights. By using an expandable structure, the visual signalling mechanisms could dynamically change their separation, for example contracting to aggregate various disparate visual signals into a cohesive display or separating to make it easier to distinguish individual visual channels from greater distances. To date, we have yet to see any work in HRI examining the use of expandable structures for such functional signalling and we believe it to be a rich, untapped area for future exploration.

#### 4.2.2 Affective Communication

Expandable structure designs are often inspired by plants and animals that increase or decrease their size as a way to escape or frighten predators. Robots equipped with expandable structures might use similar life-like patterns of expansion or contraction to convey affective information. Research has found that affective communication and giving a human-like character can improve human-robot interaction as users perceive the robot to be more intelligent (Admoni, 2016; Cha et al., 2018b). Affective communication conveys the emotions of a social robot (Hu and Hoffman, 2019) or makes information like the robot’s intent more understandable to users by exaggerating animations (Szafir et al., 2015). For example, a robot with an expandable structure that could present a danger to collocated humans or is engaged in a critical/uninterruptible task might mimic the example of the pufferfish, which increases in size when feeling threatened, by expanding to warn humans against approaching or coming near the robot. Alternatively, there are some animals that contract as a defense mechanism. For example, the leaves of the shameplant (*Mimosa Pudica*) fold inward and droop when touched or shaken, defending themselves from harm, and re-open a few minutes later. This mechanism offers an alternative inspiration for expandable structure behavior, where a similar contracting mechanism might convey weakness and robot fragility, contrasting the dangerous and intimidating nature of an expansion behavior.

Finally, expandable structure designs can also be inspired by human nature. For example, when people become anxious or afraid, their heart rate increases and they may start breathing faster. These physiological responses are a sign of discomfort and something humans may intuitively understand and feel empathy for. Alternatively, other patterns of behavior (e.g., foot tapping, skipping, etc.) are commonly associated with a variety of other affective states (e.g., irritation, joy, etc.), providing a rich area of inspiration from which HRI researchers may draw. While we believe that expandable structures hold significant promise in conveying affective information in HRI contexts, to date we have yet to see research investigating this space. We discuss our own preliminary investigations in this area in Section 5.2.

#### 4.2.3 Data Visualization

Expandable structures may also enable robots to provide new ways of visually communicating data to users. As an example, robots might use linear or radial expansion to physicalize data (e.g., forming physical bar graphs or scatterplots). One advantage that such robots may offer over static data physicalization techniques is the ability to dynamically represent data. In addition, expandable structure robots may also act as a dynamic physical displays, supporting real-time transformations of data into different representations such as bar graphs, line charts, or star graphs. We explore these aspects in Section 5.3.

### 4.3 Safety

Within the broader area of HRI, the sub-field of physical human-robot interaction (pHRI) focuses on concerns related to human safety. Several methods of ensuring physical safety have been identified in the pHRI literature, including safety through control, motion planning, prediction, and consideration of human psychological factors [see (Lasota et al., 2017) for a survey]. Specifically, with regard to physical safety, research has predominantly focused on different methods for managing collisions. Currently, most large robots that are potentially fatal to humans on collision operate only in safety cages. Other, less powerful but still potentially hazardous robots may leverage expandable structures as another form of a physical barrier. Expandable structures provide a simple, yet effective mechanism for creating dynamic boundaries around dangerous and fragile components of a robot. These structures have the potential to provide safety to three different components of any human-robot interaction scenario: the human, the robot itself, and the surrounding environment.

Many robots are comprised of various components that posses large momenta, which can result in a large impact force or pressure upon coming into contact with a human. Expandable structures provide a unique mechanism for physically separating these specific components without imposing large restrictions on robot movement or functionality. In addition to preventing collisions entirely, expandable structures also have some degree of compliance, enabling them to act as an airbag or bumper in order to reduce the impact of any collisions with a human.

Robots may also be dangerous to themselves. Certain components of robots may be fragile, such as drone propellers, or require precise and time-consuming calibration, as is the case in many industrial robots. In such cases, collisions may damage parts or shift components, requiring component replacement or recalibration. For example, the propellers of aerial robots are often extremely fragile. If a propeller comes in to contact with a surrounding object during flight, it is likely to break or deform, resulting in unstable and unpredictable flight patterns. While a static cage (i.e., propeller guard) can provide one way of protecting propellers, it permanently increases the size and shape of the robot, potentially reducing its mobility. In contrast, an expandable structure may expand to protect the propellers when the robot is more likely to collide with surrounding objects (i.e., when flying in constrained areas), and retract when not needed to give the robot more mobility.

In a similar fashion, expandable structures may reduce potential damage to any objects in the surrounding environment. For example, the compliance of expandable structures will mitigate and forces transferred from a robot to any object it hits (walls, instruments, other robots, etc.) in the event of a collision.

### 4.4 Aesthetic and Experiential Purposes

In addition to the interactive possibilities described above, expandable structures may be integrated with robotics purely for aesthetic purposes, for fun and entertainment, or to simulate users and enhance user enjoyment during the experience of working with robots. For such use, roboticists may take inspiration from aesthetic use of expandable structures in fashion (e.g., smart adaptive garments), art, or architecture. In addition, such structures might be used to give robots additional lifelike traits or quirks, such as enabling robots to mimic expansion and contraction in biological breathing movements in a manner similar to the generation of natural robot motions (Koditschek, 1984) and gaze patterns (Yoshikawa et al., 2006).

## 5 Case Studies

To advance our vision for how expandable structures may enhance HRI, in this section we detail three of our own research projects integrating expandable structures and robotics within interactive scenarios. We focus on illustrating a broad swath of the design space (e.g., different structures, robots, applications, etc.), showcasing our design and implementation process, and highlighting human responses to such robots. First, we introduce RoomShift (Suzuki et al., 2020a), a large ground robot that uses scissor-like expandable structures to move furniture in a room to enable encountered-type haptics for a human using a virtual reality headset. Next, we describe PufferBot (Hedayati et al., 2020), a medium-size aerial robot with an isokinetic expandable structure that can take several forms and afford three types of expanding behaviors. Finally, we detail ShapeBot (Suzuki et al., 2019b), a miniature tabletop robot that can alter its shape individually and as part of a larger ShapeBot swarm for a variety of purposes, including information visualization and environment manipulation.

### 5.1 RoomShift

RoomShift (Suzuki et al., 2020a) is a room-scale swarm of off-the-shelf ground robots to which we added large expandable structures to provide the robots with new environment manipulation capabilities. We then leveraged these robots to generate a new haptic feedback mechanism for virtual reality, whereby RoomShift robots reconfigure the physical environment in real time to match various virtual scenes, inspired by shelf-moving robots in robotic warehouses (Guizzo, 2008; Wurman et al., 2008).

#### 5.1.1 Design and Implementation

In their original form, each robot (a Roomba) lacks the capability to manipulate large objects. We added a mechanical lift expandable structure that can extend from 30 to 100 cm, affording the robots the ability to pick up, carry, and place objects such as chairs, tables, and movable walls. When combined with a virtual environment, the RoomShift system enables users to touch, sit, place, and lean against objects in the virtual world. In our current deployment, we have synchronized VR scenes with a 10 m × 10 m physical environment outfitted with an optical motion tracking system to support software that tracks and controls the robots. To do so, we implemented customized software in Unity which gets the user and furniture positions from the motion tracking cameras, creates the VR scene, and compiles the user’s commands to control the robots’ movement of the furniture. This system continuously maps virtual touchable surfaces in the proximity of users and coordinates the robot swarm to move physical objects to their target locations without colliding with each other or the users. The user and robots do not interact with each other directly. Since the user is fully immersed in the virtual environment, they can only see and interact with the items rendered in the VR scene (e.g., chairs, desks, etc.), which does not include the robots.

In designing RoomShift, we considered and tested several expandable structures and actuation mechanisms, including a pneumatically-actuated inflatable structure (Hammond et al., 2017; Teng et al., 2019; Suzuki et al., 2020b), a deployable structure using coilable masts (Jensen and Pellegrino, 2001; Joosten, 2007), and a mechanical structure with reel-based actuation (Takei et al., 2011b). Pneumatic actuation was problematic for our mobile setup as it requires a tube connected to a pump or pressure tank to supply air. The deployable structure and mechanical reel-based actuation afforded much higher extension ratios, but were limited in robustness and load-bearing capabilities. We finally settled on a mechanical scissor structure due to its low-cost (compact drying rack: $15, linear actuators: $35 × 2) and lightweight (2 kg) components while providing sufficient structural integrity to hold the weight of a variety of common objects. In comparison with existing warehouse robots such as Kiva (Guizzo, 2008), which have a limited expandable capability as they are designed for one specific shelf, our mechanical scissor lift can move various objects by leveraging its highly expandable structure (4× expansion ratio). The actuation height (30–100 cm) was chosen to cover a wide range of standard chairs and tables, which measure 30–76 cm and 48–96 cm, respectively (Woodworking, 2019). The maximum height of the scissor structure itself can be also extended by adding more elements, such as combining two scissor structures to double the maximum height. However, such an adjustment comes with a loss in structure stability.

#### 5.1.2 Interaction Paradigms

We deployed RoomShift in applications for supporting virtual real estate tours and collaborative architectural design, two increasingly common use cases for VR (Ibayashi et al., 2015). RoomShift supported these scenarios by enabling encountered-type haptics, whereby the robots manipulates physical objects (chairs, moveable walls, etc.) in order to adapt the physical environment to mimic the virtual user experience.

This system augmented several interaction paradigms for users (see Figure 9). For example, users could implicitly interact with the system by walking around and touching virtual objects or could explicitly interact with the virtual scene by physically moving objects tied to their virtual counterparts. Users could interact with the virtual scene with controller-based gestural interactions, for instance using controls to relocate a distant piece of furniture or removing a wall in the room. Users could also virtually teleport to new locations to navigate through virtual scenes, with RoomShift adaptively reconfiguring the physical environment to match each of the user’s new virtual locations.

**FIGURE 8:**
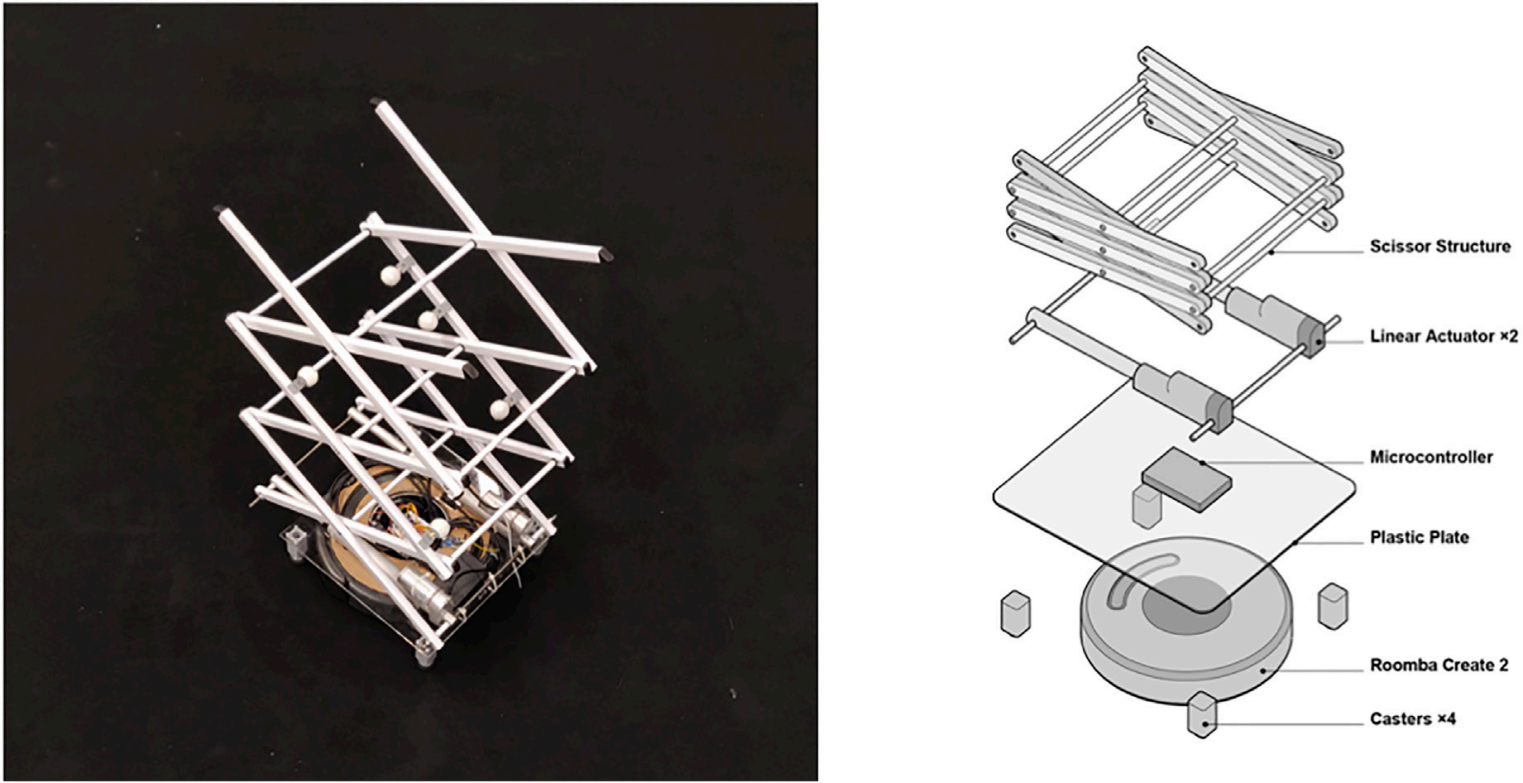
The design of RoomShift, which integrates an expandable scissor structure with a Roomba robot.

**FIGURE 9:**
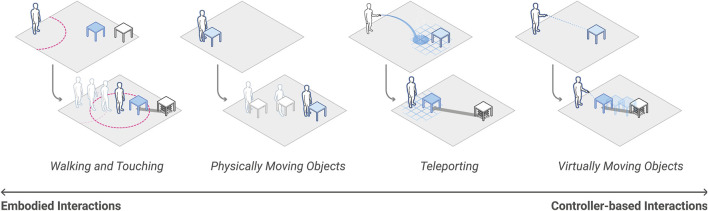
RoomShift can provide encountered-type haptics for users in a variety of VR interactions, including when users walk to touch objects, physically move virtual objects, virtually teleport to new locations, and virtually move objects.

Traditionally, a large number of physical props and robots would be required to render virtual spaces that users can walk through and touch. Instead, RoomShift leverages low-cost, expandable structures and nine off-the-shelf robots, along with the insight that a user’s immediate physical reach (e.g., ∼1.5 m radius) is usually smaller than an entire virtual scene. Therefore, the system only places haptic props within the user’s immediate proximity. As the user walks around the space, the robots move the props to maintain the illusion of a larger number of objects. In this way, a small number of robots with a finite set of physical props can suffice to provide haptics for the scene as the system does not need to physically render the entire environment.

In addition, the system can mimic larger objects with a single moving robot. For example, when the user is interacting with a large table, either new physical table segments can be added or a single robot can continually move the current table according to the user’s position to simulate touching a larger one. This way, a limited number of robots and furniture can simulate large objects. We also use this technique for rendering larger wall segments, where the robot moves, carrying the proxy, as the user walks along the wall, similar to a technique proposed in PhyShare (He et al., 2017).

RoomShift also supports scene editing within VR. The virtual scene layout editing is similar to standard VR interactions and includes functionality like adding, removing, moving, resizing, or rotating virtual building elements and furniture with a VR controller or a GUI. For example, the user can point the controller at a virtual object and move it to a target location. RoomShift robots then update the corresponding physical object’s position.

RoomShift illustrates an interesting paradigm for HRI, one in which the user does not directly interact with robots at all, but where robots seamlessly and invisibly operate in the background to augment user experiences in a manner similar to traditional goals of ubiquitous computing, where the goal of successful technologies is to fade into the background (Weiser, 1999). We conducted a preliminary evaluation of our system to gauge user responses to RoomShift (for more details on the evaluation, see (Suzuki et al., 2020a)). In a within-subjects counterbalanced design, participants interacted with physical chairs in a VR scene in two conditions: (1) with physical chairs moved by robots and (2) with static physical chairs. All participants expressed that the realism of the two conditions were the same. In general, participants indicated positive experiences and were enthusiastic about potential applications. By integrating off-the-shelf robots with inexpensive expandable structures and actuators, we added entirely new robot functionality and purpose, enabling new forms of interaction with humans working in a VR scene.

### 5.2 PufferBot

Next, we describe PufferBot (Hedayati et al., 2020), an example of how expandable structures can serve multiple purposes for robots, such as capturing human attention, conveying information, and mimicking human emotions, while also improving safety. PufferBot’s design illustrates an integration of isokinetic structures, inspired by Hoberman spheres, with aerial robots. For PufferBot, we designed four different isokinetic expandable structures (ring, cylinder, hemisphere, and sphere) and three biologically-inspired behaviors for the structure to emulate (expansion, contraction, and pulsating). Below, we detail our PufferBot design and implementation process and summarize our findings of user perceptions of PufferBot.

#### 5.2.1 Design and Implementation

Our goal in designing PufferBot was to explore the integration of expandable structures and aerial robots, with the notion that such structures might enable new forms of robot signaling and serve as protective guards to reduce the dangers of collisions. Previous robot design approaches have focused either on protecting (e.g., propeller guards) or signaling (e.g., alarms). Our insight was that expandable structures may offer a combination of both features. As a result, we identified four design constraints for the expandable structures. First, they should be low weight as additional weight may reduce robot flight time or, in the worst case, render the robot unable to fly. Second, they should be easy to build. There is a limited number of primitive shapes that can easily expand without drastically changing their structure. For example, many structures utilize spheres because the shape can expand and contract with ease. Pyramids and cubes are more rare as they are complex and less conducive to shape-changing. Third, the structures should be symmetrical, both when contracted and in the expanded shape. This is because the aerial robot’s flight controller is programmed with a predefined center of mass. Thus, the structures should not change the robot’s center of mass in the x-y plane. Changes in the z-axis however, are easier to adjust for. Fourth, we needed to design structures such that no part of the expandable structure would ever be in the way of spinning propellers, as any interaction between the propellers and the structure would lead to robot damage and likely a loss of flight.

With these constraints in mind, we designed four isokinetic structures to surround the robot: a ring, hemisphere, sphere, and cylinder (See Figure 11). The ring is a Hoberman linkage that is positioned slightly above the propellers, expanding and contracting on the x-y plane. The hemisphere consists of a ring with two orthogonal half-rings positioned above it. The sphere consists of three orthogonal rings (one oriented along the x-y plane, one along the y-z plane, and one along the x-z plane). The cylinder contains the same circle as the ring, as well as a second one positioned just below the propellers.

**FIGURE 10:**
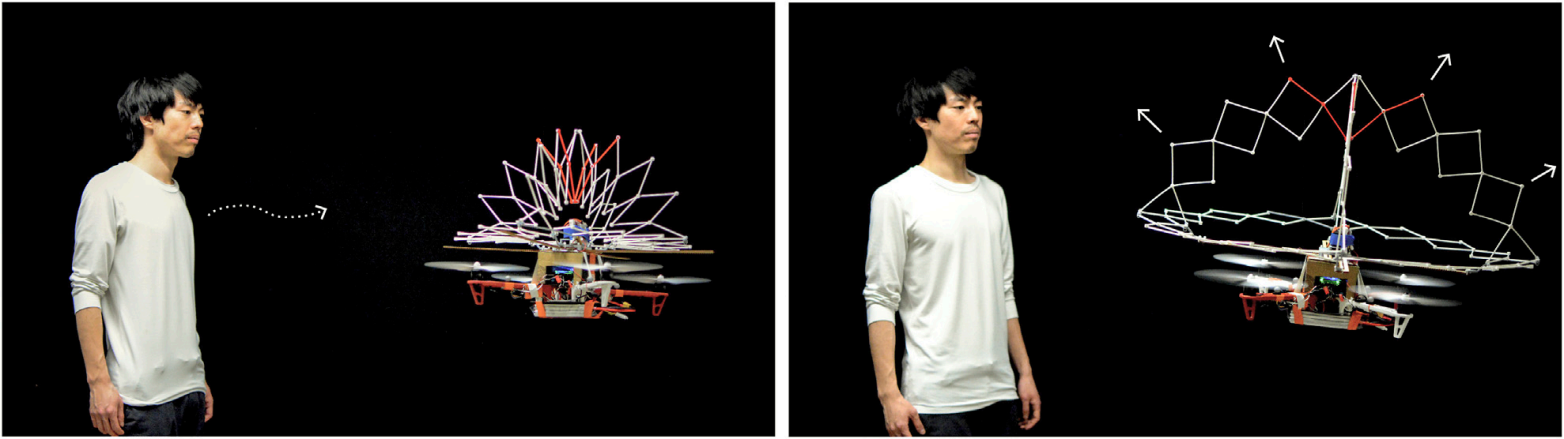
PufferBot can exhibit various communicative behaviors when humans approach the robot. Above, PufferBot expands as a user approaches to warn the human to stay away from the robot.

**FIGURE 11:**
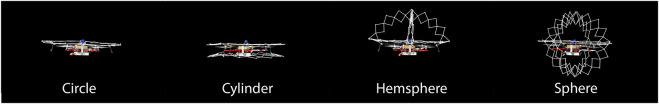
We designed four varieties of expandable structures for PufferBot, each with trade-offs in the amount of protection it can provide, visual saliency for communication purposes, and weight.

**FIGURE 12:**
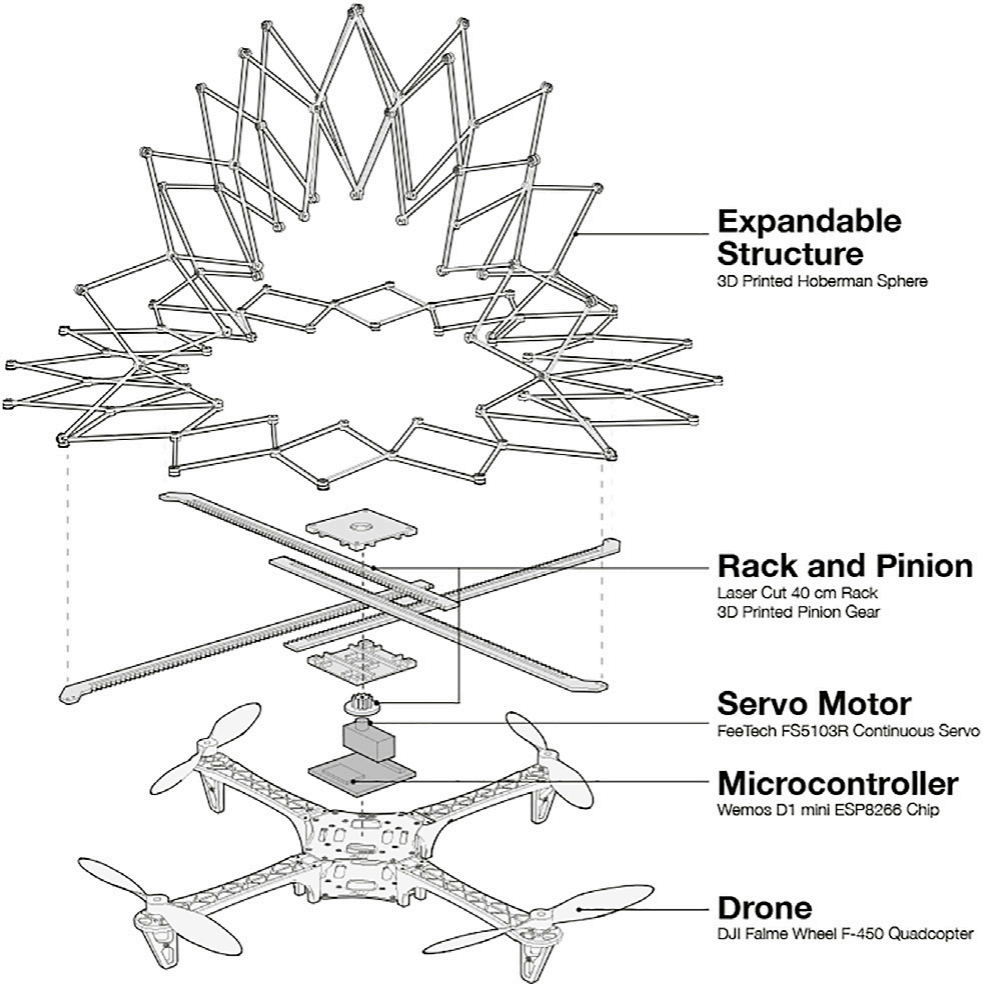
The various components of PufferBot. At the base, we use a DJI Flame Wheel F450 drone. A microcontroller is used to control the actuation mechanism, a servo motor and a rack and pinion mechanism. The racks are joined with the expandable structure to control its radius.

To implement these designs, PufferBot itself is comprised of three components: an off-the-shelf aerial robot (DJI Flame Wheel F450 frame), an electromechanical actuator, and one of the four expandable structures described above (see Figure 12). As mentioned, one of our primary concerns in designing PufferBot was structure weight. The unmodified robot frame weight is 282 g. After mounting additional components (motors, battery, flight controller, etc.), the weight of the aerial robot accumulates to 1.2 Kg. The platform itself is capable of lifting up to 1.6 Kg of payload, meaning that the expandable structure could weigh up to 0.4 Kg.

In addition, we needed to consider how to mount expandable structures to the robot frame in a manner that did not interfere with robot mobility or other internal components. The diagonal length of the robot (motor to motor) is 45 cm. We used 4.5 inch propellers (11.43 cm), which make the total length of the aerial robot 70 cm. To attach our structures, we built a plate on top of the aerial robot that provided a surface to mount and secure an expandable structure and actuator, which can be powered by the main robot power supply (we used a 4S Lithium-ion Polymer (LiPo) battery, which gives the robot a flight time of approximately 18 min). This plate also allows us to avoid direct contact with the onboard sensors in the flight controller.

We designed a one degree-of-freedom rack and pinion mechanism capable of actuating any of our four expandable structure designs. The pinion gear located in the center rotates the four individual racks at the same time, so that the actuated racks can evenly apply the expansion or contraction force to the expandable structure in four different directions with the same magnitude. The actuator joint attached to the end of the rack can expand and collapse the expandable structure by pushing and pulling the connected points. With the mounting plate and actuation mechanism, we were able to implement each of our four structure designs in a manner that satisfied all of our design constraints. In simple flight tests, we found that each of our designs could improve human and robot safety, acting in a manner similar to deployable airbags to distribute collision forces and reduce potential propeller damage (and damage caused by propellers), often enabling the robot to remain flying even after collisions.

The robot’s constraint on load capacity alongside the intended purpose of the expandable structure providing a barrier for collisions introduced a trade-off in the choice of material for the structure. While a strong, rigid structure material (e.g., metal) would provide the most protection during a collision, it would limit the allowable size of the structure, as larger structures would be too heavy for the robot to carry. On the other hand, an extremely lightweight material would be efficient in terms of load capacity, but would be more prone to break during a collision, rendering the structure ineffective. Thus, we decided to use a material that was relatively lightweight and capable of withstanding small to medium impacts and 3D printed our Hoberman linkages with PLA. We see a similar trade-off between protection and load capacity when comparing each of the four structure designs. While the sphere design offers the most protection for the robot, it is also 3 times heavier than the ring design. Similarly, the cylinder and hemisphere designs provide more protection than the ring, but are 2 times heavier.

#### 5.2.2 Exploring Communicative Behaviors

Beyond safety, we were also interested in how such structures might be leveraged as a new communicative medium in human-robot interactions. To this end, we designed three expandable structure behaviors to convey information to collocated humans, with a focus on trying to convey that users should stay a safe distance away from the robot so as to avoid creating any potentially dangerous situations. In designing these behaviors, we took inspiration from nature and how animals and plants increase or decrease their size as a way to escape or frighten predators. We describe each of the three behaviors below.

**FIGURE 13:**
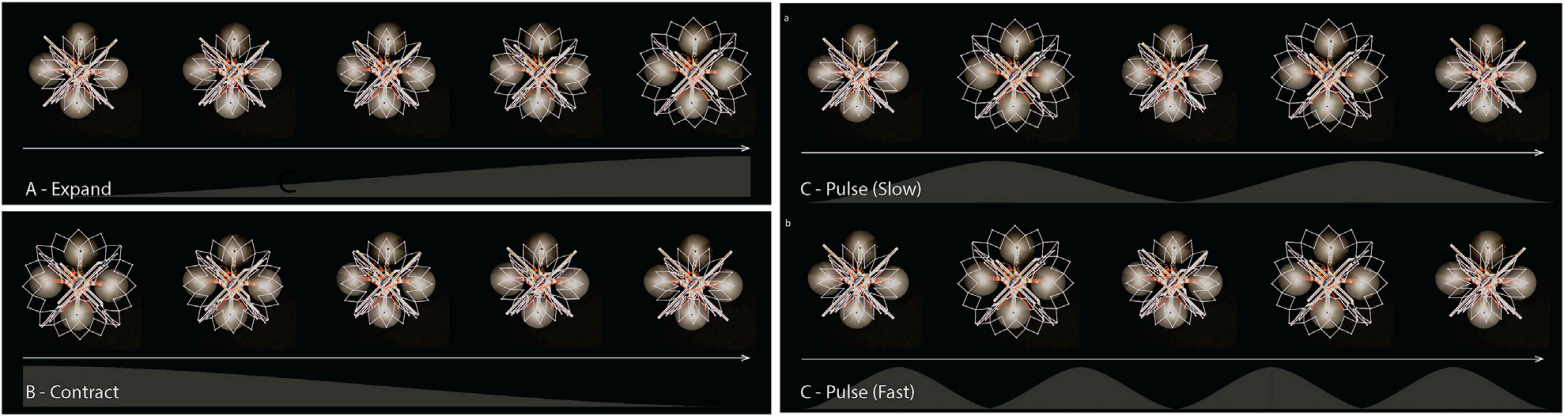
**(A)** Expand, **(B)** Contract and **(C)** Pulse behaviors.

Expand: This design is inspired by animals that expand in size when they want to frighten and scare off predators, like the pufferfish. Our expandable structure has a radius of 52 cm when contracted and expands to a radius of 82 cm in 6 seconds, covering the propellers (Figure 13A). We designed this behavior to mimic the aggressive nature of similar animal defense mechanisms, conveying a message of “don’t come near me, I’m dangerous.”

Contract: In contrast to the expand behavior, certain organisms contract as a defense mechanism. For example, the leaves of the shameplant (*Mimosa pudica*) fold inward and droop when touched or shaken, defending themselves from harm, and re-open a few minutes later. Such mechanisms inspired our second behavior (Figure 13B). During contraction, the expandable structure shrinks from an 82 cm radius to 52 cm. This behavior is intended to convey more weakness and fragility from the drone, contrasting the dangerous and intimidating nature of the expansion behavior.

Pulse: The last behavior is inspired by humans where, when a person gets anxious or afraid, their heart rate increases and they may start breathing faster. The pulse behavior consists of two sub-behaviors to mimic this physiological response. When the robot is in a room with a collocated person at a safe distance (defined as more than 3 m away (Duncan and Murphy, 2013)) the expandable structure expands and contracts at a “regular” breathing rate corresponding to approximately 20 times per minute. During this sub-behavior, the robot expands for 1 s, contracts for 1 s, and then rests for 1 s (Figure 13C-top). When the collocated person comes closer than 3 m to the robot, the robot starts to “breathe” faster: it expands and contracts within 1 second and takes no rest (Figure 13C-bottom). This is intended to indicate that the robot is anxious and the collocated person is making it uncomfortable.

We have explored people’s reactions to PufferBot’s expandable structure designs and behaviors by gathering information on user perceptions of various robot configurations through in-person demonstrations and online studies using recorded videos from multiple angles. We recruited 268 participants for this study: 260 for an online video study and eight for a follow-up, in-person study. In these studies, we focused on how PufferBot may dissuade users from approaching an interesting looking, but potentially dangerous robot, as well as how PufferBot can express emotions. For the online study, we asked participants to imagine that they were to approach the robot, upon which it would exhibit one of the three behaviors outlined above (i.e., expanding, contracting, or pulsing at a more rapid rate) with one of the structure designs, which they could see in a video. The participants then filled out a survey asking them to rate various qualities about the robot or their beliefs about it on a scale of 1–7. For the in-person study, participants were asked to approach the robot, which then executed one of the behaviors. In-person participants were shown all combinations of structures and behaviors and were asked to complete the same survey after each combination.

A common theme that we have found is that a majority of people (63% responses ≥5) believed the robot was discouraging them from approaching it when it exhibited any of the three behaviors. As a whole, the highest level of danger was conveyed by the expansion behavior (*M* = 4.42) and the hemisphere (*M* = 4.88). The highest level of anxiety was conveyed by the contract behavior (*M* = 4.92) and the hemisphere (*M* = 5.24). During an open-ended discussion with the in-person participants, some people believed that the robot was more dangerous in its contracted state, noting the greater exposure of the propellers. Similarly, some participants viewed the ring and cylinder structures to be unprotective of the robot or themselves due to the propeller exposure. It is important to note that even though only these two shapes were associated with a lack of protection, each shape exposes the propellers to some degree. Out of all combinations of structure shape and behavior, the in-person participants identified the sphere and heartbeat as being the most communicative or expressive. In general, these responses indicate that users may have complex and varied responses to our expandable structures and behaviors, although the structures in general may be capable of expressing simple states (e.g., users perceived the robot as experiencing noticeable levels of anxiety).

The open-ended responses from online participants also revealed complicated, and at times conflicting, user perspectives. Three participants believed that the intent of the structure was to protect the robot, rather than the human. For the ring, cylinder, and hemisphere structures, four participants thought the robot was signalling an intent to land. The responses below are illustrative of the diversity of participant opinions:

P47 (Hemisphere, Expand, Eye level) “It reminds me of a peacock expanding its feathers. It is trying to intimidate me, show me its strength. It is telling me to watch out.”
P136 (Sphere, Expand, Eye level): “The robot is trying to make itself more visible so I do not accidentally crash into it.”
P31 (Ring, Pulse, Below): “It almost looks like the robot is inhaling and exhaling. Like it is taking in information instead of air. As I get closer to the robot the movement seems to get faster making me believe that it is taking in more information.”
P155 (Ring, Pulse, Eye level): “It seemed to be looking around for someone more interesting than me to interact with. Maybe he’s trying to say, “you don’t interest me.”
P181 (Cylinder, Contract, eye level): “It seems to want to say ‘come here with me and follow’ to me.”
P95 (Cylinder, Pulse, Below): “It feels like the robot is extracting something from me, and since it is not physically touching me, I feel like it is trying to extract information from my phone or personal electronic devices.”

Overall, the PufferBot platform demonstrates how expandable structures and their corresponding nature-inspired behaviors might be used by robots in multiple ways simultaneously and opens the door to future research exploring the complex intersection of expandable robot structures and user responses. In the future, we hope to explore additional aspects of human-robot interaction, such as whether such structures may enhance user confidence when operating an aerial robot as crashes may cause less harm.

### 5.3 ShapeBots

As a final case study, we describe ShapeBots (Suzuki et al., 2019b), a swarm of small, self-transformable robots that can *individually* and *collectively* change their configurations to display information, actuate objects, act as tangible controllers, visualize data, and provide adaptive physical affordances. Each ShapeBot robot can change its individual shape by leveraging small linear actuators that are thin (2.5 cm) and highly extendable (up to 20 cm) in both horizontal and vertical directions. The modular design of each actuator enables various shapes and geometries of self-transformation. Below, we detail the design of ShapeBots, illustrate several potential application scenarios, and discuss how this type of interface opens up possibilities for the future of ubiquitous and distributed shape-changing interfaces for HRI.

#### 5.3.1 Design and Implementation

In contrast to RoomShift and PufferBot, where our design process involved creating expandable structures and adding them to pre-existing robot platforms, we designed ShapeBots from the ground up to be robots with embedded expandable structures. Each robot is driven by two micro DC motors (TTMotor TGPP06D-136, torque: 550 g/cm, diameter: 6 mm, length: 18 mm) that are soldered to a dual motor driver (DRV8833) and controlled by a main microcontroller (ESP8266). By individually controlling rotation speed and direction, the robot moves forward and backward and turns left and right. Two 3D printed wheels (1 cm diameter) connect directly to the DC motors. An O-ring tire on each wheel increases friction with the ground to avoid slipping. A LiPo battery (3.7 V 110mAh) powers both the microcontroller and the motors.

**FIGURE 14:**
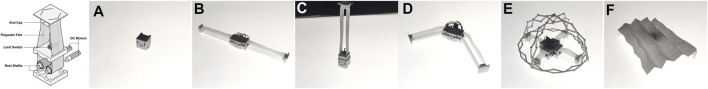
The ShapeBot expandable structure design (left) and different types of transformation it enables: **(A)** the basic ShapeBot, **(B)** horizontal extension, **(C)** vertical extension, **(D)** bending, **(E)** volume expansion, and **(F)** area expansion.

For the expandable structure, we developed a miniature reel-based linear actuator that fits into a small footprint (3 cm × 3 cm) while able to extend up to 20 cm in both horizontal and vertical directions. The modular design of each linear actuator unit enables the construction of various shapes and geometries of individual shape transformations as seen in Figure 14 (e.g., horizontal lines, vertical lines, curved lines, 2D area expansion with an expandable origami structure, and 3D volumetric change with a Hoberman mechanism). Such transformations support three major types of shape change (form, volume, and orientation) categorized in Rasmussen et al. (2012). Each robot has an additional DRV8833 motor driver to control these linear actuators; the two motor drivers connect to the microcontroller through a 2-sided custom PCB.

All components are enclosed within a 3D printed housing (3.6 cm × 3.6 cm × 3 cm) with three rectangular holes in the front side (Figure 14) that provide micro USB ports for programming, recharging, and the microcontroller reset switch. All 3D printed parts were fabricated with a FDM 3D printer (Cetus 3D MKII) and PLA filament (Polymaker PolyLite 1.75 mm True White). For horizontal extension, each linear actuator unit is fixed with a custom 3D printed holders. For the vertical extension, we used a thick double-sided tape (3M Scotch Mounting Tape 0.5 inch) on top of the swarm robot. In our current prototype, one swarm robot costs approximately 20–25 USD (microcontroller: 4 USD, motor drivers: 3.5 USD x2, DC motors: 3 USD x2, charger module: 1 USD, LiPo battery: 4 USD, PCB: 1 USD) and each linear actuator costs approximately 6-7 USD (DC motors: 3 USD x2, limit switch: 0.5 USD, polyester sheet: 0.1 USD), but this cost can be reduced with volume. For our system, we fabricated thirty linear actuator units for twelve robots. To control the swarm of robots, we implemented a custom centralized PID controller. The PID controller gets the position of ShapeBots from the unique fiducial marker attached to each of the robots using an RGB camera and sends control signals to each robot through Wifi. As an example, to create a formation (e.g., sine wave) the PID controller moves each of the robots from their current state to the desired location.

#### 5.3.2 HRI Applications

**FIGURE 15:**
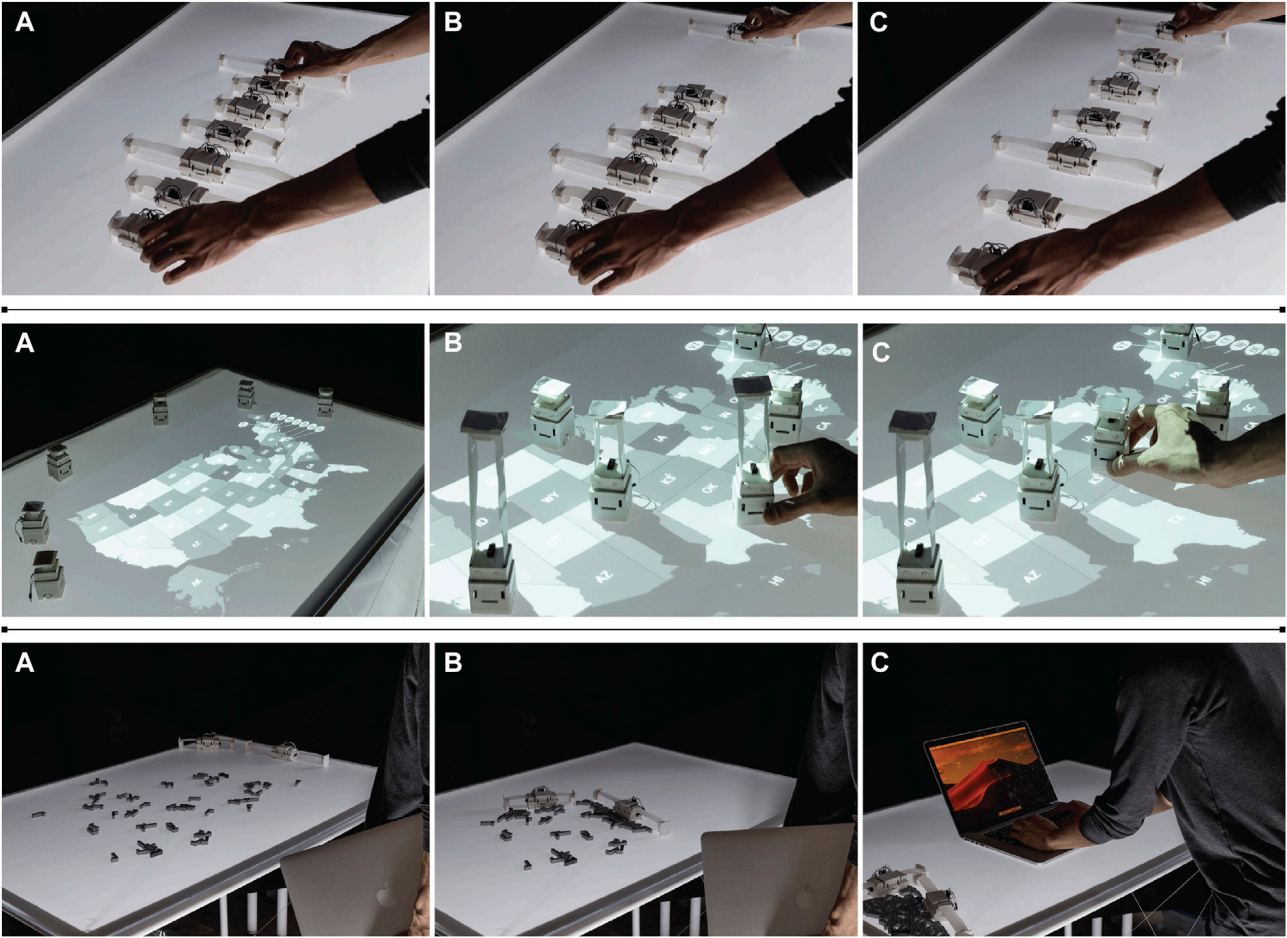
First row: An interactive and animated sine wave. **(A)** Animated sine wave. **(B)** When the user moves one element, **(C)** then each robot can collectively move to change the spatial period of the wave. Second row: Embedded data physicalization on a map. **(A)** Projected US map. **(B)** When the user selects the dataset, the ShapeBots move to position and visualize data with their heights. **(C)** When moved, the robots change their heights accordinly. Third row: Clean up robots. **(A)** A desk is filled with debris. **(B)** Two robots starts moving and wiping the debris. **(C)** Once the robots finish cleaning up, the user can start using the workspace.

We describe several application scenarios showing how a swarm of distributed self-transformable robots might support everyday interactions. For example, one potential application area is interactive data physicalization (Jansen et al., 2015; Taylor et al., 2015), as in the first row of Figure 15, where seven ShapeBots transform individually to represent a sine wave. These representations are interactive with user input: when the user moves the end robot to the right, the others move to change the wavelength. The user can dynamically change the amplitude of the wave by specifying the maximum length.

ShapeBots also support transforming data into different representations, such as bar graphs, line charts, and star graphs. Users can place and move robots, which enables embedded data representations (Willett et al., 2017). For example, ShapeBots can be placed on a map of the USA to physically represent population density by changing their height based on what state they are placed on (Figure 15, second row). Users can interact with the dataset by placing a new robot or moving a robot to a different state, with the robots updating their physical forms to represent the respective population.

Other examples of distributed representations include showing the magnitude and orientation of wind on a weather map or physicalizing magnetic force fields. This physical data representation might be particularly useful for people with visual impairments (Suzuki et al., 2017; Guinness et al., 2018). ShapeBots can also act as an interactive physical display, meaning they can render or enable users to preview various shapes. For instance, when reading a picture book of animals, children might visualize a fish with ShapeBots at actual size. Another application of Shapebots is for use as an interactive tangible display. As an example, four ShapeBots might display a small rectangle and, when the user moves a robot, the others can change positions and lengths to appropriately scale the shape. The user can also move robots to rotate or translate the shape. In this manner, ShapeBots might provide a physical preview of a CAD design (e.g., if a user is designing a box, ShapeBots can physicalize the actual size of the design). In such interactions, the design process and physical rendering are tightly coupled; as the user changes aspects of the design in CAD software, the ShapeBots change accordingly or the user can change the parameters of the design by directly moving robots in the physical space, and these changes are reflected in the CAD design. Finally, ShapeBots may provide practical assistance by their ability to actuate objects and act as physical constraints. As an example, Figure 15, third row) shows two robots extending their linear actuators to wipe debris off a table, clearing a workspace for the user.

In summary, ShapeBots are miniature tabletop robots with expandable structures that enable individual and collective shape-change. We highlight ShapeBots as an example of how robots may be designed from the beginning with expandable structures in mind and to illustrate additional collective shape-changing capabilities for human-robot interaction beyond the implicit interactions described in RoomShift.

## 6 Discussion and Future Research Directions

**FIGURE 16:**
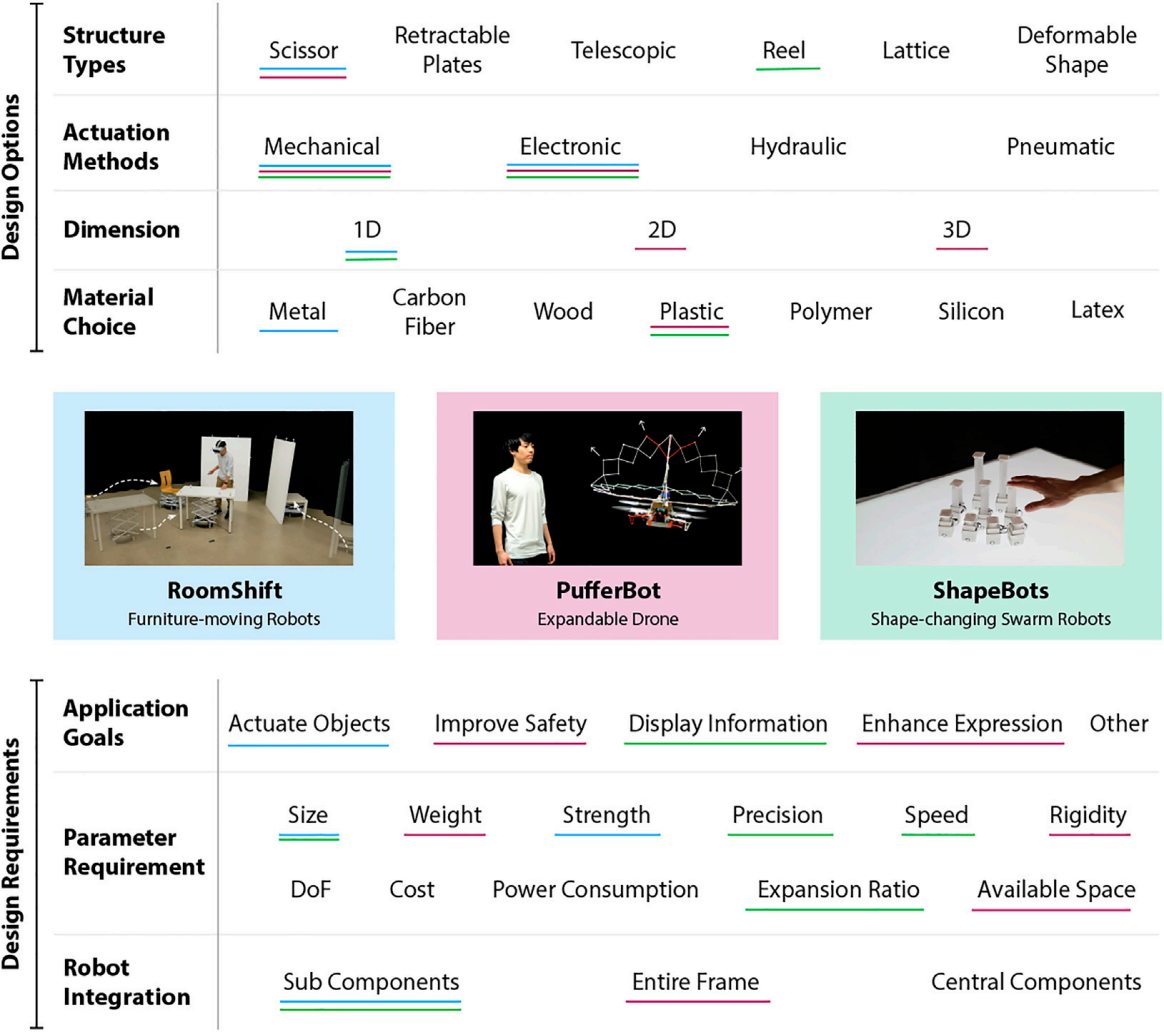
Above, we synthesize the major implementation and interaction considerations for designing expandable structures for HRI. We also highlight the choices made in each of our three case studies.

We believe that expandable structures represent a significant and underexplored avenue for HRI research. Our case studies, along with related systems such as the Triple Scissor Extender Robot Arm (Shikari and Asada, 2018) and Rotorigami (Sareh et al., 2018), demonstrate the potential of integrating of expandable structures into robotics to enrich human-robot interactions, whereby such structures may provide robots with new interactive capabilities, enable novel forms of communication, and enhance safety. In addition, we envision that such structures may serve aesthetic and experiential purposes as well, such as piquing user curiosity, increasing enjoyment, or promoting a sense of play, although we have yet to see research explore such applications of expandable structures in HRI contexts. To aid researchers and developers seeking to explore this burgeoning space, we have summarized the major design and implementation considerations for expandable structures in robotics, highlighting the choices made in our three case studies above, in Figure 16. We are excited to continue exploring expandable structures for HRI and further develop the initial design space outlined here as the research community begins to leverage expandable robots in new forms of interaction. Moving forward, we believe the following aspects hold particular promise for future research:

First, we believe research may more deeply explore the use of expandable structure robots in conjunction with virtual reality, as they show great value for augmenting VR experiences through encountered-type haptics. In contrast to RoomShift, where we used expandable structures to deliver haptic proxies, future work might investigate how expandable structures could act as various haptic proxies themselves. As the number of virtual objects that someone can interact with in a virtual world is essentially limitless, it is nearly impossible to design a system like RoomShift that can deliver any type of physical object to a user in a virtual environment unless the particular application is known in advance. However, expandable and/or shape-changing structures may be able to emulate a vast array of objects with which users can physically interact. Additionally, systems might afford users the capability of changing the physical shape or size of virtual objects while simultaneously feeling such transformations in their hands.

Next, we envision future work may investigate how expandable structure robots might improve users’ wellness and productivity. Through our work with PufferBot, we have found that expandable structures may alter the various anthropomorphic emotions and personality traits that humans naturally ascribe to robots. We would like to explore how to leverage expandable structures to change human perceptual responses to robots in a principled manner and believe that the range of possibilities is much greater than the small subset of affective traits we have explored to date. For example, future work might examine how expandable structure robots could convey emotions such as empathy or tranquility to improve user wellness or visualize aesthetically pleasing objects, such as blooming flowers, to bring joy to users. As a practical example, expandable structures with behaviors similar to the pulse pattern exhibited by PufferBot might be used as a guide for breathing patterns, as is done in meditation practice, in a robot-guided meditation interaction. Towards improving users’ productivity, we are interested in how small expandable robot like Shapebot that could integrate within user workstations might help users visually keep track of schedules, provide appointment reminders, or increase user motivation through emotive expressions.

Beyond individual interactions, we anticipate that expandable structure robots may also hold benefits for interacting with crowds. For instance, robots with expandable structures might be used to create dynamic boundaries around areas, which could change size depending on the size of the crowd. On a larger scale (e.g., crowds of thousands of people), we envision that a swarm of robots with expandable structures might be used to direct crowd movement, such as providing guidance towards exits or along evacuation routes, by expanding to block incorrect or overcrowded paths and marking available routes. In emergency scenarios, robots might also leverage expandable structures to create space for injured parties or protect privacy.

Ultimately, we envision a future where shape-changing technologies have been woven into standard robot design practices, enabling robots to dynamically adapt to users and their environment. Expandable structures can play a key role in this vision by serving as low-cost, easy-to-implement, and easy-to-control methods to augment robot capabilities. We believe the time is ripe for HRI research to examine their potential for enhancing human-robot interactions. We hope the design space and case study examples provided here will help advance and encourage further research in this area.

## Footnotes

1 Such structures are also commonly described as “deployable.” As we are primarily focused on the use of such structures with robotics to improve HRI, in this paper we use the terminology of “expandable” to avoid potential confusion with the notion of “deploying” robots for particular applications.
